# Predicting Mismatch-Repair Status in Rectal Cancer Using Multiparametric MRI-Based Radiomics Models: A Preliminary Study

**DOI:** 10.1155/2022/6623574

**Published:** 2022-08-16

**Authors:** Guodong Jing, Yukun Chen, Xiaolu Ma, Zhihui Li, Haidi Lu, Yuwei Xia, Yong Lu, Jianping Lu, Fu Shen

**Affiliations:** ^1^Department of Radiology, Changhai Hospital, Shanghai, China; ^2^Department of Radiology, Ruijin Hospital Luwan Branch, Shanghai Jiaotong University School of Medicine, Shanghai, China; ^3^Huiying Medical Technology Co., Ltd., Beijing, China; ^4^Department of Radiology, Ruijin Hospital, Shanghai Jiaotong University School of Medicine, Shanghai, China

## Abstract

Detecting mismatch-repair (MMR) status is crucial for personalized treatment strategies and prognosis in rectal cancer (RC). A preoperative, noninvasive, and cost-efficient predictive tool for MMR is critically needed. Therefore, this study developed and validated machine learning radiomics models for predicting MMR status in patients directly on preoperative MRI scans. Pathologically confirmed RC cases administered surgical resection in two distinct hospitals were examined in this retrospective trial. Totally, 78 and 33 cases were included in the training and test sets, respectively. Then, 65 cases were enrolled as an external validation set. Radiomics features were obtained from preoperative rectal MR images comprising T2-weighted imaging (T2WI), diffusion-weighted imaging (DWI), contrast-enhanced T1-weighted imaging (T1WI), and combined multisequences. Four optimal features related to MMR status were selected by the least absolute shrinkage and selection operator (LASSO) method. Support vector machine (SVM) learning was adopted to establish four predictive models, i.e., Model_T2WI_, Model_DWI_, Model_CE-T1WI_, and Model_combination_, whose diagnostic performances were determined and compared by receiver operating characteristic (ROC) curves and decision curve analysis (DCA). Model_combination_ had better diagnostic performance compared with the other models in all datasets (all *p* < 0.05). The usefulness of the proposed model was confirmed by DCA. Therefore, the present pilot study showed the radiomics model combining multiple sequences derived from preoperative MRI is effective in predicting MMR status in RC cases.

## 1. Introduction

Rectal cancer (RC) represents a major gastrointestinal malignancy worldwide, with steadily increasing incidence and death rates [1–3]. To date, immune checkpoint inhibitors (ICIs) have become a crucial therapeutic option for improving prognosis in several solid tumors [4, 5]. Previous clinical trials have shown that microsatellite instability (MSI) and/or mismatch-repair deficiency (dMMR) constitute significant tissue-agnostic molecular markers for the prediction of ICIs' efficacy [6–9]. Because genetic and immunohistochemical (IHC) tests for MMR deficiency are available, pembrolizumab and nivolumab as monotherapies or combined with ipilimumab have had approval from the US Food and Drug Administration (FDA) for treating chemoresistant MSI/dMMR mCRC cases [10].

Accurate prediction and diagnosis of MMR status in patients with RC is important in designing a treatment plan and prognostic evaluation. Although laboratory genetic testing and tissue biopsy have been applied to assess the amounts of MMR proteins in RC, including MLH1, MSH2, MSH6, and PMS2, these approaches are costly, invasive, and/or time-consuming [11, 12]. More importantly, since different parts of the tumor could have distinct MMR expression levels, MR imaging may better capture this heterogeneity as a whole rather than a needle biopsy of a single tumor component.

Currently, radiomics, a novel noninvasive tool, has been widely used for pretreatment assessment as well as treatment outcome, distant metastasis, and local recurrence predictions in RC, providing important details of tissue characteristics inaccessible to human eyes [13–16]. The radiomics approach was inspired by the notion that medical images comprise considerable information reflecting potential pathophysiological properties through quantitative analysis of digital medical images for the whole tumor. Since not all patients are subjected to genomic tests, radiogenomics is vital because individuals may undergo imaging examinations during the course of disease. Therefore, radiomics data originated from the complete tumor rather than only a tissue sample and could provide gene expression or mutation data to increase diagnostic, predictive, and prognostic capabilities, enabling precision therapy [17–20]. However, the prognostic and predictive value of MRI-based radiomics for evaluating MMR status preoperatively in RC still deserves further attention.

Therefore, this study focused on the radiomics features of RC, aimed at assessing the value of radiomics models derived from multiparametric MR imaging for preoperatively predicting MMR status.

## 2. Materials and Methods

### 2.1. Patients

The current trial had approval from the Committee on Ethics of Changhai Hospital and Ruijin Hospital Luwan Branch, Shanghai, China. Informed consent was not required because of the retrospective design.

Pathologically confirmed RC cases administered rectal MRI and surgical resection in Changhai hospital from January 2018 to December 2019 were enrolled into the training and test sets. Next, individuals meeting the above eligibility criteria in Ruijin Hospital Luwan Branch were enrolled from January to December 2020 into the validation set (external validation cohort).

**Figure 1:**
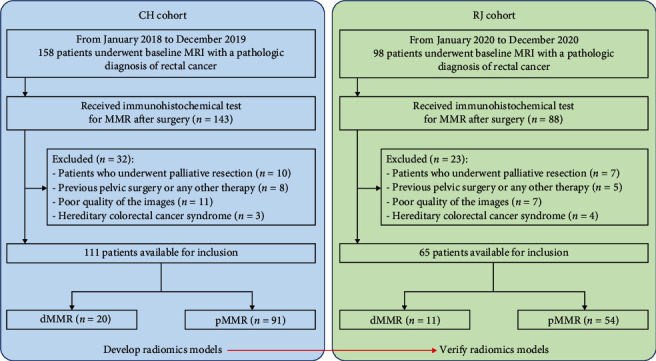
Study flowchart. CH Cohort: Changhai Hospital; RJ Cohort: Ruijin Hospital Luwan Branch.

Inclusion criteria were as follows: (1) MRI with a pathologic diagnosis of RC, (2) baseline MRI exam within 2 weeks before surgical resection, (3) immunohistochemical test for MMR after surgery, and (4) single focal lesion. Exclusion criteria were as follows: (1) palliative resection; (2) previously administered pelvic surgery, radiation therapy, chemotherapy, or chemoradiotherapy; (3) image quality unsuitable for tumor segmentation; and (4) hereditary colorectal cancer syndrome. Totally, 111 and 65 patients were eventually included in the Changhai and Ruijin cohorts, respectively (Figure 1). Then, the random number technique (random seeds = 24) was carried out for assigning 70% and 30% of the cases in the Changhai cohort to the training and test sets, respectively.

Baseline clinical information was collected, including age, gender, BMI, presurgical carcinoembryonic antigen (CEA) and carbohydrate antigen (CA19-9) levels, and distant metastasis. An experienced radiologist (G.J.), with 10 years of experience, obtained the data from medical records.

### 2.2. Image Acquisition

After fasting for 4 hours, the patients were administered enema with 20 ml of glycerin prior to MR scanning. Raceanisodamine hydrochloride was not utilized because of potential contraindications.

Routine rectal MRI sequences were carried out on a 1.5 T or 3.0 T MR scanner, i.e., oblique axial high-resolution T2WI without fat suppression, sagittal T2WI, axial diffusion-weighted imaging (DWI; *b* value = 0; 1000 s/mm^2^), axial T1-weighted imaging (T1WI), and gadolinium contrast-enhanced T1WI (CE-T1WI) in the sagittal, coronal, and axial planes. CE-T1WI scans were obtained at 1 min following Gd-DTPA (Beilu Pharmaceutical, China) injected intravenously at 2 ml/s with a high-pressure syringe and saline flush (20 ml at 2 ml/s). Details regarding the parameters applied for the above sequences are listed in Supplemental Table 1.

### 2.3. Pathological Evaluation

Mismatch-repair (MMR) status was determined based on surgical specimens, confirmed by immunohistochemical staining of four MMR proteins, including MLH1, MSH2, MSH6, and PMS2. Deficiency in any of these proteins was defined as dMMR. Based on the National Comprehensive Cancer Network and American Joint Committee on Cancer (AJCC) TNM system (8th Edition) [21], two pathologists with more than 10 years of work experience determined the tumor's TN stage, histological type, differentiation status, tumor deposit, lymphovascular invasion, perineural invasion, tumor budding, and circumferential resection margin (CRM) from hematoxylin and eosin- (H&E-) stained slices. In case of discrepancy, both examiners discussed to reach a consensus.

### 2.4. Image Segmentation

The original DICOM images underwent importation into the Radcloud radiomics platform (Huiying Medical Technology, China). As MR image acquisition utilized distinct MRI systems in both hospitals, the images were normalized for homogeneity using the following formula:

(1) $$\begin{array}{c} f\left(x\right)=\frac{s\left(x-\mu_{x}\right)}{\sigma_{x}}, \end{array}$$

where *f*(*x*) is the normalized intensity, *x* is the original intensity, *μ*_*x*_ is the mean of the image intensity value, *σ*_*x*_ is the standard deviation of the image intensity value, and *s* is an optional scaling, by default, which is set to 1.

Regions of interest (ROIs) in all RC cases were manually delineated slice-by-slice along the clearest solid border that best fitted the lesion area, excluding the blurry margin, on each of these three sequences (T2WI, CE-T1WI, and DWI with *b* = 1000 s/mm^2^). Then, volumes of interest (VOIs) were derived from the obtained ROIs. All images were processed by 2 experienced radiologists (Z.L. and H.L., with 11 and 6 years of experience in abdominal imaging, respectively) in an independent manner, blinded to group assignment. All segmentations were checked by one senior radiologist (F.S., who had 12 years of experience in rectal MRI).

### 2.5. Radiomics Feature Selection

Based on the derived whole VOI segmentations, radiomics feature extraction was performed with the above platform from T2WI, DWI, CE-T1WI, and combined sequences, respectively. Four types of features were obtained: (1) first-order statistics, including peak and mean value (with variance), quantifying the distribution of voxel intensities on MR scans; (2) shape properties, including volume, lesion area, and spherical value, reflecting the 3D features of the delineated area's shape and size; (3) texture features, i.e., gray-level cooccurrence, run length, size zone, and neighborhood gray-tone difference matrices, quantitating a given area's heterogeneity; and (4) higher-order statistics, encompassing transformed first-order statistics and texture features [13–15], e.g., logarithm, gradient, square, and wavelet transform.

In the training cohort, inter- and intraclass correlation coefficients (ICCs) were determined to assess feature robustness. Features with inter- and intraobserver ICCs above 0.8 were further analyzed. Next, the variance threshold. Select-*K*-best and least absolute shrinkage and selection operator (LASSO) algorithms were employed to choose optimal parameters. In addition, the synthetic minority oversampling technique (SMOTE) was utilized to tackle the imbalanced samples in the training cohort for subsampling. The SMOTE algorithm represents an improved sampling strategy, with a given novel synthetic sampling computed according to the Euclidian distance for variables.

### 2.6. Radiomics Model Establishment

**Figure 2:**
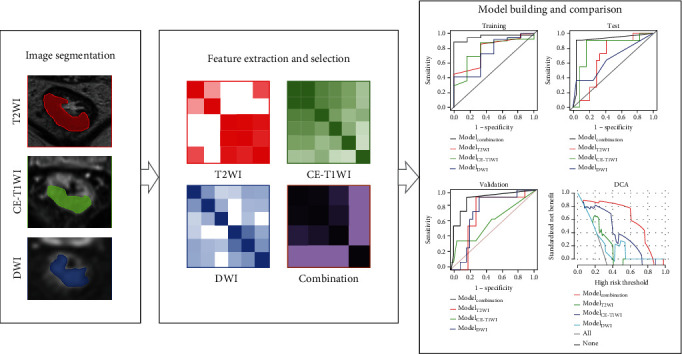
Workflow for building the radiomics model.

The Radcloud platform was utilized for SVM learning with the “scikit-learn” package in Python (v0.24.1, https://scikit-learn.org/stable/). Based on the optimal features related to MMR status, the SVM with linear kernel was employed to construct 4 predictive models: (1) Model_T2WI_, (2) Model_DWI_, (3) Model_CE-T1WI_, and (4) Model_combination_, with optimal features extracted from T2WI, DWI, and CE-T1WI sequences in combination. To prevent overfitting, the validation set was employed for verifying and comparing the performances of the final models.  Figure 2 depicts the radiomics workflow. Furthermore, the penalty coefficient with the best performance was used to train the final SVM model.

### 2.7. Statistical Analysis

Continuous variables were assessed for normality by the Kolmogorov-Smirnov test, and group comparisons were performed by the *t*-test or the Wilcoxon test. Categorical variables were compared by the Chi-square or Fisher's exact test. Variance threshold methods were applied for selecting radiomics features (variance threshold = 0.8), taking out eigenvalues below 0.8. For the select-*K*-best method, utilizing *p* values for analyzing the associations of radiomics features with MMR status, features with *p* < 0.05 were used. For the LASSO algorithm, L1 regularizer was used as the cost function, and optimal *λ* value was derived based on the minimum of the average mean square error by 5 cross-validation and 1000 iterations. Radiomics features with a nonzero coefficient in LASSO were chosen by linearly combining the chosen features multiplied by the corresponding coefficients for each patient. Receiver operator characteristic (ROC) curve analysis was carried out for performance evaluation for each model by deriving the area under the ROC curve (AUC) and determining sensitivity, specificity, and accuracy. ROC curve comparisons utilized the DeLong test. Decision curve analysis (DCA) was applied to assess the benefits of each model. The nomogram was analyzed with R v3.6.3. Other data were analyzed with SPSS 20.0 (SPSS, USA) and MedCalc 19.6.1. *p* < 0.05 was deemed statistically significant.

## 3. Results

### 3.1. Patient Features

Totally, 111 and 65 individuals in the Changhai and Ruijin cohorts were finally enrolled, respectively.  Table 1 lists the patient features, which had similar characteristics in both cohorts (Supplemental Table 2). Based on MMR status determined by postsurgical pathological assessment, 20/111 individuals (18.0%) were categorized as dMMR in the Changhai cohort, versus 11/65 (16.9%) in the Ruijin cohort (*p* = 0.854). Subsequently, 78 (70.3%) and 33 (29.7%) cases in the Changhai cohort were assigned to the training and test sets, respectively.

### 3.2. Radiomics Features

**Figure 3:**
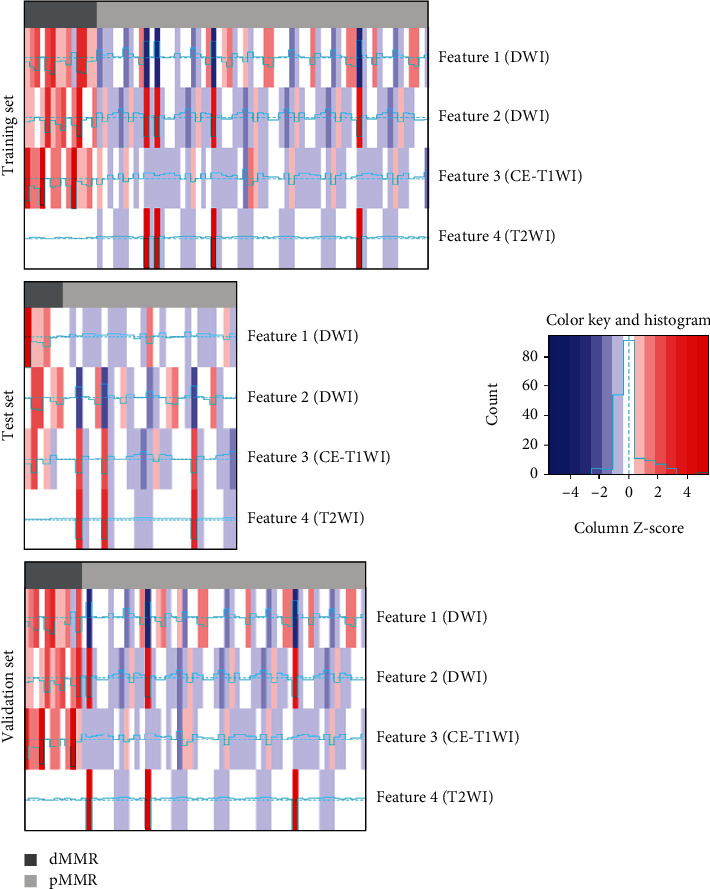
Heat map shows the distribution of radiomics features between the dMMR and pMMR groups. Each row in the heat map corresponds to a radiomics feature, each column corresponds to one patient. dMMR: deficient mismatch repair; pMMR: proficient mismatch repair.

Totally 1409 radiomics features were obtained from T2WI, DWI, and CE-T1WI data. Totally, 1270/1409 (90.1%), 1232/1409 (87.4%), and 1268/1409 (90.0%) of them had inter- and intraobserver ICCs above 0.8, respectively, from T2WI, DWI, and CE-T1WI, and were further examined. Totally, 429, 466, and 406 features were selected in subsequent variance threshold and the select-*K*-best algorithm, respectively. Eventually, two, five, and ten optimal features associated with MMR status were determined with the LASSO algorithm from T2WI, CE-T1WI, and DWI data, respectively (Supplemental Table 3). Then, the combination of T2WI, CE-T1WI, and DWI resulted in four screened features from 3770 features for predicting MMR status. Details are presented in Table 2. A heat map showed the discrepant distribution of the selected features between the dMMR and pMMR groups (Figure 3).

### 3.3. Radiomics Models

**Figure 4:**
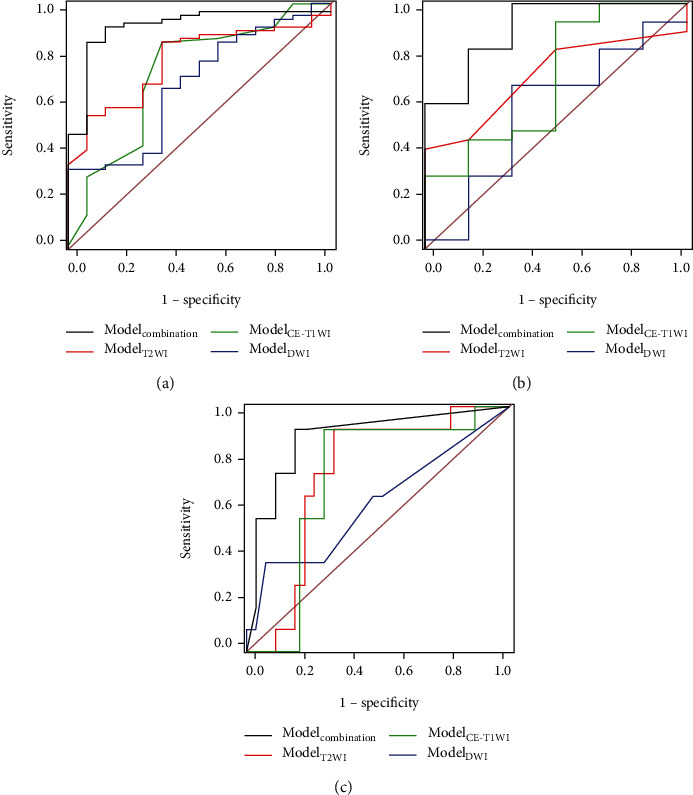
ROC curves for the SVM models. The performance of Model_combination_ was better than those of other models (*p* < 0.05). (a) In the training set. (b) In the test set. (c) In the validation set.

In the training population, Model_T2WI_, Model_DWI_, Model_CE-T1WI_, and Model_combination_ had diagnostic performances reflected by AUCs between 0.670 and 0.910 (Figure 4(a)). Model_combination_ achieved the best diagnostic performance (AUC = 0.910; accuracy = 85.9%). In the test set, the four models had diagnostic performances reflected by AUCs between 0.568 and 0.901 (Figure 4(b)). Model_combination_ had the best performance (AUC = 0.901; accuracy = 93.9%).

While validating the radiomics models, Model_combination_ had the best diagnostic performance (AUC, 0.874; sensitivity, 90.9%; specificity, 81.5%; accuracy, 83.1%) in the validation set (Figure 4(c)). These findings indicated Model_combination_ had improved discrimination performance in comparison with other models (*p* < 0.05) in all datasets.  Table 3 presents the detailed findings.

### 3.4. Decision Curve Analysis

**Figure 5:**
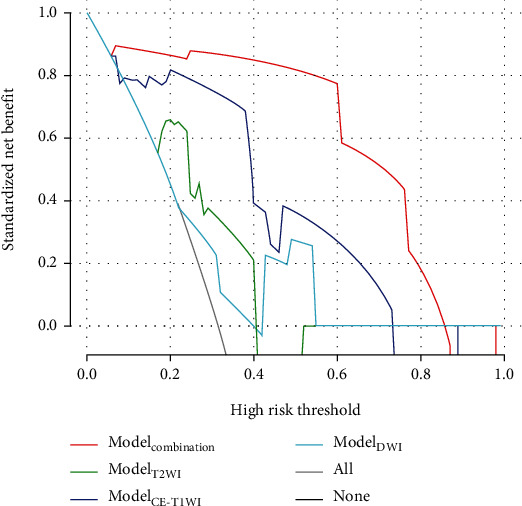
Decision curve analysis of the prediction model in the validation set. The light gray line represents the assumption that all patients had dMMR. The dark gray line represents the hypothesis that no patients had dMMR. The red curve shows that with the probability of MMR ranging from 0.06 to 0.86, using the radiomics Model_combination_ to predict MMR of RC would be beneficial than other radiomics models.

DCA demonstrated an adequate performance for Model_combination_ in distinguishing dMMR lesions from pMMR counterparts in the validation cohort. Model_combination_ had clinical superiority over the other models for net benefit within a large threshold probability (Figure 5), suggesting the multiparametric MRI approach had significantly improved power in comparison with other models.

## 4. Discussion

Here, a machine learning model based on the combination of multiple MRI sequences constituted an effective, noninvasive, novel imaging approach for evaluating MMR status in RC cases, with an external validation set examined by different MRI scanning equipment and conditions.

High microsatellite instability (MSI) results from malfunction of the dMMR system and is responsible for about 3–5% metastatic colorectal cancers (mCRCs) [22–25]. Detecting MMR in rectal cancer is important in clinical decision making, identifying individuals with differential treatment response and prognosis. Studies have shown that standard nCRT might be less effective in dMMR RC patients than in patients with proficient mismatch repair (pMMR) [24]. It might lead to the modification of clinical practice in avoidance of unnecessary nCRT with poor response and dMMR can be used as a biomarker to guide clinical immunotherapy.

Although it is recommended to perform neoadjuvant CRT for most patients with locally advanced RC according to the NCCN guideline. However, in clinical practice, determination of whether to receive perioperative CRT was at the discretion of the surgeon, oncologist, and patient. If CRT was not performed before operation, the postoperative CRT could be considered. The management and treatment strategies could be tailored after identification of LARC patients who would benefit from adjuvant therapy or are not likely to exhibit a good response to nCRT, if MMR status was detected in the pretreatment approach.

Universal immunohistochemical testing for evaluating MMR status is recommended. However, due to this relatively costly and time-consuming approach, some patients remain untested [26]. Therefore, broadly available, low-cost, and noninvasive methods are urgently needed to help select patients for evaluation. More importantly, since different parts of the tumor could have distinct MMR expression levels, imaging approach may better capture this heterogeneity as a whole rather than a needle biopsy of a single tumor component. This study investigated a machine learning-based model for automatically predicting MMR directly from MR images.

In comparison with routine strategies using imaging methods, radiomics substantially improves disease diagnosis, tumor grading, and prognostic evaluation, providing a comprehensive guidance for treatment planning [16–18]. A previous study demonstrated that the SVM model showed good classification performance related to pathological features in patients with RC [13]. With continuous technology progress, the concept of “radiogenomics” has been widely applied in tumors recently. Via extraction of multiple quantitative parameters from imaging findings combined with genomics data, and performing a deep mining of associations between both data types, radiogenomics can be used to retrieve quantitative image information that can reflect gene expression for deeper understanding of the occurrence and development of tumors, through noninvasive, conventional imaging methods [27].

Currently, several studies have reported the correlation between radiomics features and MSI status in colorectal cancer based on CT images [28–31]. Meanwhile, only limited recently published studies have developed MRI-based radiomics models for predicting MSI status preoperatively in rectal cancer [32, 33]. Although the latter reports found that radiomics models show great potential in predicting MSI status, there is currently no correlation study with external validation between MMR prediction and multiparametric MRI-based radiomics in RC.

The most valuable aspect of the present study is the multiparametric approach that enhances the MRI-based radiomics model by mining complementary information provided by multiparametric MRI and considering the heterogeneity of tumors for predicting differential features involved in MMR status [33]. By extracting radiomics features hardly detectable visually from the preoperative MR scans of the segmented VOIs of whole primary tumors, we developed four predictive models with T2WI sequence alone, DWI sequence alone, CE-T1WI sequence alone, and the combination of these three sequences, respectively. Model_T2WI_ contained phenotypic features, while Model_DWI_ and Model_CE-T1WI_ contained heterogeneous data describing microcirculation for the entire rectal tumor. Heat map analysis revealed a correlation between MMR status and features, suggesting the chosen multiparametric features had relevance to MMR status preferably.

The combined radiomics model achieved the overall best performance in predicting MMR status among all models in both cohorts, showing superiority over single-sequence models. Good clinical utility was demonstrated by decision curve analysis. Combining many MRI sequences and deep mining of correlations among distinct radiomics features could allow a comprehensive assessment of tumor heterogeneity, which might increase predictive efficiency and potentially guide in distinguishing cases who need individualized treatment.

The second vital aspect of this study is that we had an actual external validation dataset, adding value to existing reports. Machine learning models raise high concern for overfitting. Using an external cohort is very helpful for overcoming the weakness that the developed model has no exposure to a validation cohort in the training phase in any form.

However, this project is still in its infancy, with many limitations. First, an important limitation of the current retrospective trial was its relatively small sample size and unbalanced distribution. This implies selection bias and low generalizability of the results, although we used an external validation cohort and the SMOTE algorithm to reduce the effect of unbalanced sample distribution. Consequently, large multicenter studies are warranted for reducing the effects of selection bias on model accuracy. Secondly, the imaging segmentation approach was manual rather than automatic, which may suffer from subjective errors and could be unsuitable for data processing in case of large sample size [34, 35]. Thirdly, a study previously developed and validated deep learning models for the prediction of MSI status in RC based on MRI data [36]. In future research, deep learning model with feature map may show more advantages over other approaches to visualize heterogeneous distribution. It provides a possibility that the deep learning can be used to predict which tumor area is most likely to show dMMR to guide biopsy.

## 5. Conclusions

Overall, based on preoperative rectal MRI, the established multiparametric machine learning model demonstrated good performance in predicting MMR status in RC patients. This radiomics approach could better the current strategy for the pretreatment of patients, with the advantage of being noninvasive and cost-effective, potentially helping select patients suitable for individualized therapy.

## Figures and Tables

**Table 1:** Demographic and pathological data of the patients in both cohorts.

Variables	CH cohort		*p* value	RJ cohort		*p* value
	dMMR (*n* = 20)	pMMR (*n* = 91)		dMMR (*n* = 11)	pMMR (*n* = 54)	
Gender (male/female)	16/4	56/35	0.117	7/4	36/18	1.000
Age (year)	55.8 ± 9.9	57.1 ± 10.8	0.775	59.2 ± 11.9	60.1 ± 11.4	0.881
BMI (kg/m^2^)	23.2 ± 3.1	23.9 ± 3.8	0.660	24.1 ± 4.0	23.5 ± 3.5	0.747
Histological type						
Adenocarcinoma	13	73	0.238	7	41	0.639
Mucinous adenocarcinoma	7	18		4	13	
Pathological T stage						
T1-2	7	32	0.989	4	20	1.000
T3-4	13	59		7	34	
Pathological N stage						
N0	8	35	0.898	5	25	0.959
N1-2	12	56		6	29	
Clinical M stage						
M0	4	26	0.434	3	16	1.000
M1	16	65		8	36	
Tumor location						
Upper	3	8	0.700	2	10	0.589
Middle	11	53		8	32	
Lower	6	30		1	12	
Differentiation						
Well	3	14	0.213	1	10	0.330
Moderate	14	46		8	26	
Poor	3	31		2	18	
Tumor deposit						
No	13	62	0.786	7	28	0.475
Yes	7	29		4	26	
Lymphovascular invasion						
No	12	47	0.498	8	29	0.408
Yes	8	44		3	25	
Perineural invasion						
No	12	63	0.425	5	30	0.540
Yes	8	28		6	24	
Tumor budding						
No	11	66	0.124	6	35	0.764
Yes	9	25		5	19	
KRAS						
Wild type	12	67	0.223	6	33	0.946
Mutant type	8	24		5	21	
NRAS						
Wild type	13	58	0.915	8	30	0.473
Mutant type	7	33		3	24	
BRAF						
Wild type	11	52	0.861	7	28	0.475
Mutant type	9	39		4	26	
CEA^∗^						
<5 ng/ml	14	52	0.289	8	32	0.619
≥5 ng/ml	6	39		3	22	
CA19-9^∗^						
<37 U/ml	15	77	0.480	10	39	0.354
≥ 37 U/ml	5	14		1	15	

CH cohort: Changhai Hospital, training and test sets; RJ cohort: Ruijin Hospital Luwan Branch, validation set; BMI: body mass index; dMMR: deficient mismatch repair; pMMR: proficient mismatch repair; CEA: carcinoembryonic antigen; CA19-9: carbohydrate antigen 19-9. ^∗^Postoperative blood samples.

**Table 2:** Comparisons of selected features between different MMR status.

No.	Radiomics feature	Sequence	dMMR median (interquartile range)	pMMR median (interquartile range)	*Z* value^†^	*p* value	Coefficients^∗∗^
1	Wavelet-LLH^∗^_ GLSZM_ zone variance	DWI	5.131 (4.378-5.300)	3.388 (2.950-3.968)	3.521	<0.001	-0.075
2	Wavelet-HLH^∗^_ GLSZM_ zone variance	DWI	6.244 (5.800-8.000)	5.000 (4.350-5.200)	2.312	0.021	-0.059
3	Wavelet-HLH^∗^_ GLSZM_ large area high gray level emphasis	CE-T1WI	4458.739 (3441.205-5718.022)	2524.119 (2098.914-3682.686)	2.505	0.012	-0.052
4	Gradient_ first order_ kurtosis	T2WI	175982.000 (92672.000-213782.500)	85442.833 (31509.800-107171.778)	2.955	0.003	-0.032

GLSZM: gray level size zone matrix. ^∗^The wavelet transform decomposes the tumor area image into low-frequency components (L) or high-frequency components (H) in the *x*, *y*, and *z* axes. ^†^Mann–Whitney test. ^∗∗^The coefficients in LASSO algorithm.

**Table 3:** ROC analysis of the radiomics models.

		Model_T2WI_	Model_DWI_	Model_CE-T1WI_	Model_combination_
Training set	AUC	0.768	0.670	0.718	0.910
	95% CI	0.659-0.856	0.554-0.772	0.605-0.814	0.823-0.963
	Sensitivity	0.844	0.328	0.844	0.844
	Specificity	0.643	1.000	0.643	0.929
	Accuracy	0.808	0.449	0.808	0.859
	NRI^∗^	0.285	0.444	0.285	/
	*p* value^∗^	0.028	0.043	0.002	/

Test set	AUC	0.707	0.568	0.691	0.901
	95% CI	0.523-0.852	0.385-0.738	0.507-0.840	0.746-0.977
	Sensitivity	0.407	0.667	0.926	1.000
	Specificity	1.000	0.667	0.500	0.667
	Accuracy	0.515	0.667	0.848	0.939
	NRI^∗^	0.260	0.334	0.241	/
	*p* value^∗^	0.019	0.042	0.042	/

Validation set	AUC	0.721	0.603	0.702	0.874
	95% CI	0.595-0.825	0.474-0.722	0.576-0.809	0.768-0.943
	Sensitivity	0.909	0.364	0.909	0.909
	Specificity	0.667	0.926	0.704	0.815
	Accuracy	0.708	0.831	0.738	0.831
	NRI^∗^	0.148	0.434	0.111	/
	*p* value^∗∗^	0.004	0.025	0.001	/

Model_combination_: based on the combination of multisequences. ^∗^NRI: net reclassification improvement, Model_combination_ compared with other models. ^∗∗^Compared with Model_combination_ by DeLong test.

## Data Availability

The datasets used and/or analyzed during the current study are available from the corresponding author on reasonable request.

## Abbreviations

RC: Rectal cancer
DWI: Diffusion-weighted imaging
MRI: Magnetic resonance imaging
T1WI: T1-weighted imaging
T2WI: T2-weighted imaging
VOIs: Volumes of interest
ROC: Receiver operating characteristic
AUC: Area under the ROC curve
SVM: Support vector machine
FOV: Field of view
BMI: Body mass index
CEA: Carcinoembryonic antigen
CA19-9: Carbohydrate antigen 19-9
dMMR: Deficient mismatch repair
pMMR: Proficient mismatch repair
DCA: Decision curve analysis
SMOTE: Synthetic minority oversampling technique
LASSO: Least absolute shrinkage and selection operator.

## Supplementary Materials

Supplementary 1 Supplemental Table 1: main sequences and parameters of rectal MRI.

Supplementary 2 Supplemental Table 2: characteristics of the two examined patient cohorts.

Supplementary 3 Supplemental Table 3: selected radiomics features for different sequences.
