# Supplementary material for: Predicting Mismatch-Repair Status in Rectal Cancer Using Multiparametric MRI-Based Radiomics Models: A Preliminary Study
Source: Biomed Res Int. 2022 Aug 16;2022:6623574. doi: 10.1155/2022/6623574 (PMC9400426; doi:10.1155/2022/6623574)
Supplement: Supplementary 1 — Supplemental Table 1: main sequences and parameters of rectal MRI. [file 6623574.f1.docx]

**Supplemental Table 1.** The main sequences protocol and parameters of rectal MRI

| **Cohort** | **CH Cohort** | | | **RJ Cohort** |
| --- | --- | --- | --- | --- |
| **Scanner** | **GE**  **Discovery 750w** | **GE**  **Signa HDX** | **SIEMENS**  **Magnetom Skyra** | **SIEMENS**  **Magnetom Avanto** |
| **Magnetic field strength** | 3.0T | 3.0T | 3.0T | 1.5T |
| **Oblique axial T2WI** |  |  |  |  |
| Echo train length | 32 | 14 | 16 | 15 |
| Field of view (mm) | 200X200 | 180X180 | 180X180 | 180X180 |
| Section thickness (mm) | 4 | 3 | 3 | 3 |
| Matrix | 352X352 | 288X288 | 320X320 | 320X320 |
| TR/TE (ms) | 6538/116 | 4000/113 | 4000/108 | 4000/90 |
| Bandwidth (kHz) | 62.5 | 83.3 | 108/Pixel | 182/Pixel |
| Flip angle (°) | 110 | 90 | 160 | 150 |
| **Axial contrast enhanced T1WI** |  |  |  |  |
| Field of view (mm) | 340X340 | 330X330 | 300X243.9 | 380X308.9 |
| Section thickness (mm) | 4 | 5 | 4.5 | 3 |
| Matrix | 256X224 | 320X256 | 288X201.6 | 320X217.6 |
| TR/TE (ms) | 4/Minimum | 1/Minimum | 5.9/2.5 | 4.7/2.8 |
| Bandwidth (kHz) | 142.9 | 166.7 | 390/pixel | 400/pixel |
| Flip angle (°) | 12 | 12 | 9 | 10 |
| **Axial DWI** |  |  |  |  |
| Field of view (mm) | 320X256 | 350X350 | 380X380 | 380X285 |
| Section thickness (mm) | 6 | 6 | 5 | 6 |
| Matrix | 128X128 | 130X96 | 150X150 | 128X128 |
| TR/TE (ms) | 2840/Minimum | 4500/Minimum | 6300/89 | 4000/75 |
| b values (s/mm^2^) | 0, 1000 | 0, 1000 | 0, 1000 | 0, 1000 |

TR/TE: Repetition time/echo time.
