# Supplementary material for: Predicting Mismatch-Repair Status in Rectal Cancer Using Multiparametric MRI-Based Radiomics Models: A Preliminary Study
Source: Biomed Res Int. 2022 Aug 16;2022:6623574. doi: 10.1155/2022/6623574 (PMC9400426; doi:10.1155/2022/6623574)
Supplement: Supplementary 2 — Supplemental Table 2: characteristics of the two examined patient cohorts. [file 6623574.f2.docx]

**Supplemental Table 2.** Characteristics of the two cohorts

| **Variables** |  | **CH Cohort** | **RJ Cohort** | ***P* value** |
| --- | --- | --- | --- | --- |
|  |  | **(n=111)** | **(n = 65)** |  |
| Gender (Male / Female) |  | 72/39 | 43/22 | 0.862 |
| Age (years) |  | 56.9±10.4 | 59.0±11.5 | 0.216 |
| BMI (kg/m^2^) |  | 23.8±3.3 | 23.5±3.7 | 0.579 |
| Histological type | Adenocarcinoma | 86 (77.5%) | 48 (73.8%) | 0.585 |
|  | Mucinous adenocarcinoma | 25 (22.5%) | 17 (26.2%) |  |
| Pathological T stage | T1-2 | 39 (35.1%) | 24 (36.9%) | 0.811 |
|  | T3-4 | 72 (64.9%) | 41 (63.1%) |  |
| Pathological N stage | N0 | 43 (38.7%) | 30 (46.2%) | 0.335 |
|  | N1-2 | 68 (61.3%) | 35 (53.8%) |  |
| Clinical M stage | M0 | 30 (27.0%) | 19 (29.2%) | 0.753 |
|  | M1 | 81 (73.0%) | 46 (70.8%) |  |
| Tumor location | Upper | 11 (9.9%) | 12 (18.5%) | 0.097 |
|  | Middle | 64 (57.7%) | 40 (61.5%) |  |
|  | Lower | 36 (32.4%) | 13 (20.0%) |  |
| Differentiation | Well | 17 (15.3%) | 11 (16.9%) | 0.956 |
|  | Moderate | 60 (54.1%) | 34 (52.3%) |  |
|  | Poor | 34 (30.6%) | 20 (30.8%) |  |
| Tumor deposit | No | 75 (67.6%) | 35 (53.8%) | 0.070 |
|  | Yes | 36 (32.4%) | 30 (46.2%) |  |
| Lymphovascular invasion | No | 59 (53.2%) | 37 (56.9%) | 0.628 |
|  | Yes | 52 (46.8%) | 28 (43.1%) |  |
| Perineural invasion | No | 75 (67.6%) | 35 (53.8%) | 0.070 |
|  | Yes | 36 (32.4%) | 30 (46.2%) |  |
| Tumor budding | No | 77 (69.4%) | 41 (63.1%) | 0.391 |
|  | Yes | 34 (30.6%) | 24 (36.9%) |  |
| KRAS | Wild type | 79 (71.2%) | 39 (60.0%) | 0.128 |
|  | Mutant type | 32 (28.8%) | 26 (40.0%) |  |
| NRAS | Wild type | 71 (64.0%) | 38 (58.5%) | 0.468 |
|  | Mutant type | 40 (36.0%) | 27 (41.5%) |  |
| BRAF | Wild type | 63 (56.8%) | 35 (53.8%) | 0.708 |
|  | Mutant type | 48 (43.2%) | 30 (46.2%) |  |
| CEA^*^ | <5 ng/ml | 66 (59.5%) | 40 (61.5%) | 0.786 |
|  | >= 5ng/ml | 45 (40.5%) | 25 (38.5%) |  |
| CA19-9^*^ | < 37U/ml | 92 (82.9%) | 49 (75.4%) | 0.229 |
|  | >= 37U/ml | 19 (17.1%) | 16 (24.6%) |  |
| MMR status | dMMR | 20 (18.0%) | 11 (16.9%) | 0.854 |
|  | pMMR | 91 (82.0%) | 54 (83.1%) |  |

CH Cohort: Training and test sets.

RJ Cohort: Validation set.

BMI: body mass index.

dMMR: deficient mismatch repair; pMMR: proficient mismatch repair.

CEA: carcinoembryonic antigen; CA19-9: carbohydrate antigen 19-9.

^*^ Postoperative blood samples.
