# Supplementary material for: Predicting Mismatch-Repair Status in Rectal Cancer Using Multiparametric MRI-Based Radiomics Models: A Preliminary Study
Source: Biomed Res Int. 2022 Aug 16;2022:6623574. doi: 10.1155/2022/6623574 (PMC9400426; doi:10.1155/2022/6623574)
Supplement: Supplementary 3 — Supplemental Table 3: selected radiomics features for different sequences. [file 6623574.f3.docx]

**Supplemental Table 3.** Selected radiomics features of different sequences

| **Sequence** | **No.** | **Radiomics feature** | **Radiomics class** | **Filter** |
| --- | --- | --- | --- | --- |
| **T2WI** | 1 | busyness | NGTDM | wavelet-LLL^*^ |
|  | 2 | run length nonuniformity | GLRLM | exponential |
| **CE-T1WI** | 1 | maximum | first order | wavelet-HLL^*^ |
|  | 2 | range | first order | wavelet-HLL^*^ |
|  | 3 | total energy | first order | wavelet-HLL^*^ |
|  | 4 | high gray level zone emphasis | GLSZM | wavelet-LLH^*^ |
|  | 5 | size zone nonuniformity | GLSZM | wavelet-LHL^*^ |
| **DWI**  **(b=1000 s/mm^2^)** | 1 | large area low gray level emphasis | GLSZM | wavelet-LHL^*^ |
|  | 2 | zone variance | GLSZM | wavelet-LLH^*^ |
|  | 3 | kurtosis | first order | wavelet-HHL^*^ |
|  | 4 | kurtosis | first order | square |
|  | 5 | maximum 3D diameter | shape | original |
|  | 6 | maximum 2D diameter slice | shape | original |
|  | 7 | small area high gray level emphasis | GLSZM | wavelet-HHH^*^ |
|  | 8 | run length nonuniformity | GLRLM | wavelet-LHH^*^ |
|  | 9 | run length nonuniformity | GLRLM | wavelet-HHH^*^ |
|  | 10 | large area low gray level emphasis | GLSZM | wavelet-HLH^*^ |

GLSZM = Gray level size zone matrix.

GLDM = Gray Level dependence.

GLRLM = Gray level run length matrix.

NGTDM = Neighborhood gray tone difference matrix.

^*^The wavelet transform decomposes the tumor area image into low-frequency components (L) or high-frequency components (H) in the x, y, and z axes.
